# The costs and logistics of distributing ‘forest packs’ containing novel vector control tools to forest-exposed populations in Cambodia

**DOI:** 10.1186/s12936-024-05237-x

**Published:** 2025-01-07

**Authors:** Joshua Yukich, Dyna Doum, David J. McIver, Jason H. Richardson, Siv Sovannaroth, Neil F. Lobo, Allison Tatarsky

**Affiliations:** 1 School of Public Health and Tropical Medicine, Tulane University, New Orleans, USA; 2 Health Forefront Organization, Phnom Penh, Cambodia; 3 Malaria Elimination Initiative, Institute for Global Health Sciences, University of California San Francisco, San Francisco, USA; 4 Innovative Vector Control Consortium (IVCC), Liverpool, UK; 5 Cambodia National Centre for Parasitology, Entomology, and Malaria Control, Phnom Penh, Cambodia; 6 Eck Institute for Global Health, University of Notre Dame, South Bend, USA

**Keywords:** Malaria, Vector-control, Cost, Spatial repellent, Topical repellent, Treated clothing, Forest, Economic evaluation

## Abstract

**Background:**

Malaria incidence in the Greater Mekong Subregion has been on the decline, and most remaining malaria risk in the region is concentrated among hard-to-reach populations, especially those with exposure to forested areas. New vector control tools focused on outdoor protection in forest settings are needed for these populations.

**Methods:**

The delivery of a ‘forest pack’ containing a volatile pyrethroid spatial repellent (VPSR), a topical repellent, and pyrethroid treatment of clothing was evaluated in an operational study in Cambodia. Costs were collected using micro-costing approaches and the cost of distribution for the ‘forest pack’ was estimated using standard economic evaluation approaches and examined in sensitivity analyses.

**Results:**

The cost per eligible person (the target population) per malaria season for the whole pack was estimated to be 138 USD, which was nearly entirely driven by the cost of the products.

**Conclusions:**

Modifications to the ‘forest pack’ including adding a longer-lasting spatial repellent product or a reduced-cost topical repellent could significantly reduce the cost of pack distribution over the course of a malaria season.

## Background

Malaria incidence in the Greater Mekong Subregion has been on the decline for the past decade due to active control and elimination programmes as well as environmental changes [1]. As a result, risk of malaria tends to be heavily concentrated in hard-to-reach populations, many of whom work or live in forested or forest fringe areas [2–5]. Mosquito vector populations are also very active in forested areas, preferring to bite outdoors and throughout the day and night [6]. Given overlapping human and vector behaviours, domicile-focused vector control strategies, such as insecticide-treated bed nets (ITNs) and indoor residual spraying (IRS), are likely to have a limited impact on the remaining malaria risk [2, 5–9]. New vector control tools are needed for these populations.

One potential approach to protecting individuals at high risk for malaria is delivery of bite prevention tools including topical repellents, spatial repellents and insecticide treatment of clothing, which can address current gaps in protection [10–16].

Combining these tools into one single package which can be sold or distributed to at-risk populations in a ‘forest pack’ offers an opportunity to test the acceptability, use, and feasibility of these products in real-world applications. This study is part of a larger research programme called Project BITE (Bite Interruption Toward Elimination), which aims to evaluate several novel vector control tools that are targeted specifically to individuals at high-risk of malaria in outdoor settings. Project BITE evaluated the entomological efficacy of the tools in semi-field [17] and field settings [18], as well as user acceptability [19], feasibility, willingness to pay [20], and cost.

To date there are no published studies of the cost of distribution of combined bite prevention interventions to forest exposed populations in Southeast Asia. This manuscript reports on the costs of distribution of a package of bite prevention tools including a volatile pyrethroid spatial repellent (VPSR), a topical repellent, and pyrethroid-based insecticidal treatment of clothing to forest-exposed populations in Cambodia.

## Methods

### Intervention description

**Fig. 1:**
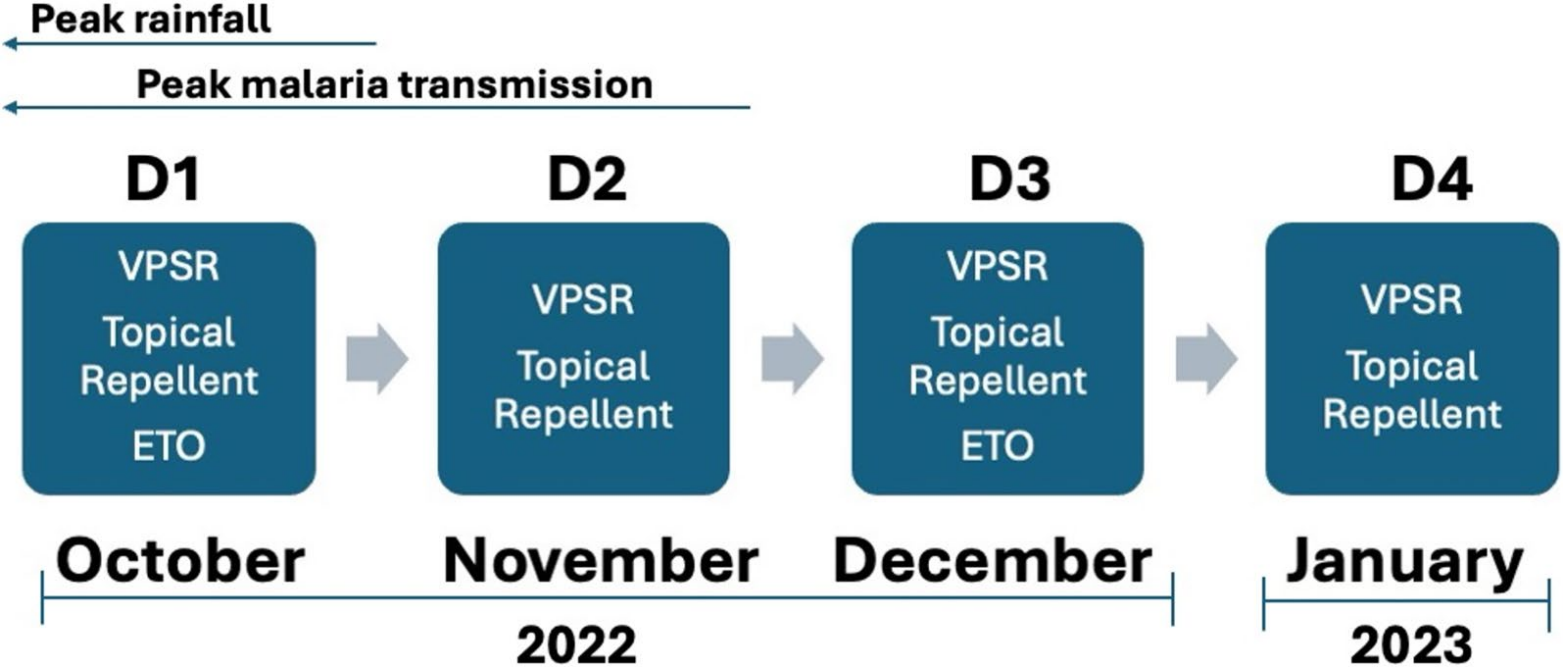
BITE Forest Pack Distribution Schedule and Components. D: distribution; ETO: etofenprox solution for clothing treatment; VPSR: volatile pyrethroid spatial repellent

An intervention description was compiled by reviewing BITE project documents and reports and conducting key-informant interviews with key project stakeholders about the project implementation. Forest packs contained two units of topical repellent (based on calculations of estimated usage per day and volume of product per unit), two units of VPSR (to be used, as per manufacturer’s instructions, simultaneously based on volume of living structures and residual efficacy of 30 days), and an etofenprox solution applied by VMWs and project staff for the treatment of clothing. BITE forest packs were delivered by VMWs in both provinces with oversight from Cambodia National Centre for Parasitology, Entomology, and Malaria Control (CNM) in Kampong Speu and an NGO (Malaria Consortium) in Mondulkiri. Packs were distributed monthly, for four months, beginning in October 2022 and ending in January 2023 (Fig. 1). The first and third distributions included forest packs which contained all three interventions, while the second and fourth did not include etofenprox solution for treating clothing. The spray-on etofenpox solution for clothing is effective for up to 25 washes (based on manufacturer’s data), which was estimated to cover a period of at least two months based on data from a previous formative assessment with the target populations [19] so only bi-monthly treatment was required.

### Study sites and target population

The study took place in both Mondulkiri and Kampong Speu provinces, two provinces reporting *Plasmodium falciparum* hotpots at the time of planning. The target population in each province were individuals who were exposed to forested settings, whether through economic activities (incl. hunting, foraging, growing/harvesting crops, logging) or non-economic activities (such as travelling from community to community through forested areas). BITE forest packs were distributed between October and January to coincide with the rainy season in Cambodia, which overlaps with the peak malaria season (Fig. 1).

### Cost and resource use data collection and analysis

Cost data were collected for the distribution of BITE forest packs in Cambodia. Costing followed a micro-costing approach, meaning that where possible, an ingredients approach was utilized, and the price and quantity of all inputs were estimated. Where this information was not available, line-item aggregated expenditures were utilized directly. The cost analysis takes the provider perspective and estimates the gross cost of BITE forest pack deployment.

Both financial costs (expenditures attributable to the provider of the intervention) and economic costs (a measure of the resources used by the provider of the intervention representing the opportunity costs of those resources) were quantified. Capital items included wholly owned vehicles, warehouses, offices, and sprayers for the application of insecticide. Costs for capital goods (those with useful lifetimes longer than one year) were annuitized using an item-specific lifetime and a global discount rate or treated as rented items and valued based on an appropriate rental cost. All annuitization assumed a 3% discount rate. BITE forest pack products were valued at approximate wholesale prices.

All costs were converted to 2022 US dollars (USD) by first converting them from the recorded currency to USD using an annual average exchange rate for the period in which the cost was incurred and then inflating them to 2022 USD, where necessary, using the US gross domestic product (GDP) deflator [21].

### Outputs and outcomes

Data on the population of the study sites, the populations considered eligible for forest pack distribution, and the number of packs (or components thereof) distributed were recorded during each distribution round. Unit cost results are presented in terms of cost per eligible (targeted) person per round, cost per pack distributed, and cost per resident person-year.

### Base case scenario

Under the base case scenario, all forest pack products were assumed to have been used consistently with the reporting from the project, and returned unused products were assumed to not incur resource costs except for their transportation. Product prices for donated topical repellents were assumed based on picaridin-based topical repellents. All product prices were assumed to be wholesale price points for valuation. The discount rate was assumed to be 3% and clothing for pyrethroid treatment was assumed to be provided at no cost to the provider by beneficiaries (*i.e.*, beneficiaries’ personal clothing was treated). Assumptions used in base case scenario and sensitivity analysis are included in Table 1.

**Table 1:** Basic inputs and assumptions including sensitivity (USD 2022)

Parameter	Input
Price of VPSR	6 USD (per unit)^*^
Price of Etofenprox	20 USD (per 950 ml bottle)
Price of topical repellent	12 USD [per unit (bottle)]^*^
Discount rate	3%
Price of VPSR (high)	8 USD (per unit)
Price of VPSR (low)	4 USD (per unit)
Duration of VPSR (long)	4 months (full season)
VPSR only scenario	One round of distribution only required [most distribution costs variable by round (except SBCC development and office space)], no sprayers required

^*^Two units included in each forest pack

### Sensitivity analysis

One-way sensitivity and scenario analyses were conducted to assess the implications of cost-model assumptions on the estimated unit costs of the intervention. These assumptions included the discount rate, the prices of products, use/uptake of pyrethroid treatment of clothing, the duration of spatial repellent product life (30 days vs. > four months), and the free provision of clothing for pyrethroid treatment by the participants to the programme. A scenario analysis was conducted to focus on the delivery of a single long lasting spatial repellent product that could last for an entire season. Additional scenario analyses were conducted to assess the costs of delivering the components of the forest packs as individual products and thus to assess potential cost savings due to integration of delivery as a forest pack unit.

### Budget impact analysis

Cost of delivering the interventions was coupled with estimates of the eligible populations in a single operational district in each province (Kampong Speu and Mondulkiri) to make estimates of the gross budget implications of scaling the intervention to the full eligible population in these districts under the base-case scenario and in alternative delivery scenarios. Estimates of the eligible populations were generated under a population size estimation study as part of the BITE research programme [22]

## Results

### Intervention description

#### Forest packs

Forest packs consist of a supply of three bite prevention products: (1) VPSR, made of a synthetic material impregnated with transfluthrin (BiteBarrier, manufactured by PIC Corporation); (2) topical repellent, an aerosol 20% picaridin formulation (Autan, manufactured by SC Johnson), and (3) pyrethroid treatment of clothing, wherein a diluted solution of etofenprox and water (Perimeter ETO Insect Guard, manufactured by Pine Belt Processing, Inc.) is applied to participant-provided clothing via a small, handheld sprayer.

Forest packs consisted of two units of VSPR, two units (bottles) of topical repellent, and an etofenprox solution for treating personal clothing, although the etofenprox solution was delivered separately as described below. At the time of this study, one VPSR unit consisted of two sheets of synthetic material approximately the size of two A4 pieces of paper. The VPSR is a passive intervention—each unit is meant to be hung approximately 5 ft off the ground, on opposite sides of a room or living structure. Once hung, the VPSR continues to work for a period of at least 30 days. Two units of the topical repellent were provided in each forest pack to provide coverage for a 30 day period. The etofenprox solution for treatment of clothing was applied via a small, handheld sprayer by a village malaria worker (VMW) or village leader—rather than directly by individual participants—to clothing that was provided by participants. The VMWs or other health or village representatives would spray up to five articles of individuals’ personal clothing. Forest packs, along with social and behaviour change communication materials, were delivered to individuals on a monthly basis by VMWs with oversight from government and NGO implementation partners (*i.e.*, packs were not provided to end-users by the research staff). Forest packs were provided for a four-month period, covering most of the high malaria transmission season.

#### Sensitization

Initial community sensitization meetings were undertaken to provide briefings to community leaders, representatives of the provincial health departments and operational districts, health centre staff, village malaria workers (VMWs) and mobile malaria workers (MMWs), village chiefs, other local authorities, and forest rangers. The meetings involved communication about the study, the three bite prevention tools to be included in the forest packs, and malaria risk, prevention, and treatment.

#### Distribution

Seventeen villages and five forest-ranger stations in Mondulkiri province were selected for inclusion in this costing study. Distribution of forest packs took place between October 2022 and January 2023 in four rounds spaced approximately one month apart. Full forest packs (including pyrethroid treatment of clothing) were distributed in Rounds 1 and 3 while in Rounds 2 and 4 only VPSR and topical repellents were distributed. Distribution evolved over time, shifting from a central village-level distribution towards a house-to-house approach. Health centre staff and VMWs/MMWs were engaged as distributors in village settings while forest ranger stations received direct deployment from project staff.

Over the course of the four rounds of distribution, a total of 10,492 complete packs (an average of 2623 packs per distribution) were delivered to a population of around 13,000 persons, of whom approximately 4000 were considered eligible for direct receipt of the intervention due to forest-related risk factors. This amounts to a coverage rate of, on average, 87.4% of the targeted eligible population per distribution round. Complete distribution results are shown in Table 2. A more detailed analysis of metrics related to coverage will be presented in another Project BITE manuscript.

**Table 2:** Distribution results for the BITE forest pack in Mondulkiri, Cambodia

Round	VPSR (units)	Topical repellents (units)	Etofenprox treatments (persons)	Etofenprox treatments issued (bottles)	Complete packs delivered	Incomplete packs delivered	Pyrethroid treatments recovered (Bottles)	Total population	Estimated eligible population
Round 1	7000	7000	1979	–	1979	1,521	–	13,173	4137
Round 2	6992	6992	0	–	3493	3	–	13,266	4137
Round 3	7008	7008	1457	–	1457	2,047	–	13,262	4133
Round 4	7000	7000	0	–	3500	0	–	13,262	4133
Total	28,000	28,000	3436	730	10,429	3,571	459	13,262	4133

### Cost results

The total costs of the distribution programme in the base case scenario are shown in Table 3. These costs are heavily dominated by product costs (nearly 90% of the total). Aside from products, only personnel costs remain a major cost driver in the programme. Of the product costs, VPSR and topical repellents represent the largest cost contributors while pyrethroid treatment of clothing with etofenprox is much less costly, in part due to having fewer treatments delivered than planned.

**Table 3:** Distribution costs for BITE forest pack (base case scenario, USD 2022)

Line item	Capital	Cost	Percentage
Personnel	No	29,247.18	5.1%
Vehicles	Yes	4573.37	0.8%
Office/warehouse	Yes	831.50	0.1%
Sprayers	Yes	50.00	0.0%
Supplies	No	1659.54	0.3%
Other charges	No	542.02	0.1%
SBCC material development	No	18,679.46	3.3%
SBCC printing	No	6635.00	1.2%
Topical repellent	No	336,000.00	58.8%
VPSR	No	168,000.00	29.4%
Etofenprox	No	5420.00	0.9%
Total		571,638.07	100.0%

The unit cost estimates with various denominators are shown in Table 4. The cost per person (total population) is estimated to be 43.10 USD and cost per person (in eligible/targeted population) is estimated to be 138.31 USD. Population numbers relate to the cost per distribution of packs relative to the total and eligible populations for four rounds (months) of distribution. Product prices included in the BITE pack in base case scenario analysis were 6.00 USD for each unit of VPSR (two per pack), 12.00 USD per topical repellent unit (two per pack) and 20 USD per 950 mL bottle of concentrated etofenprox for treatment of clothing.

**Table 4:** Unit costs for BITE forest pack (base case scenario)

Product cost included	Cost per complete pack delivered	Cost per incomplete pack delivered	Cost per person (in total population)	Cost per person [in eligible (targeted) population]
Yes	54.81	160.08	43.10	138.31
No	5.97	17.42	4.69	15.05

### Sensitivity analysis

**Fig. 2:**
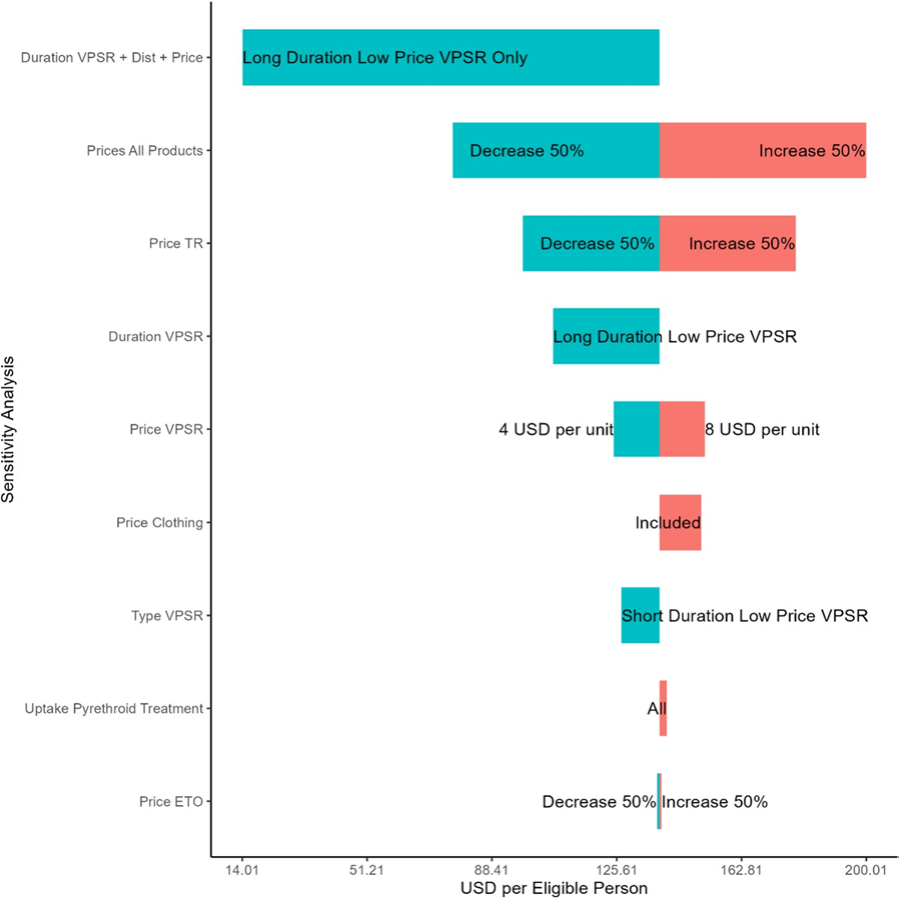
Tornado Plot of sensitivity analysis results

One-way sensitivity and scenario analyses were conducted to determine the impact of various assumptions on the unit costs for the distribution of the BITE forest pack. Product input prices for VPSR and topical repellents were major drivers of the unit cost of the distribution as a whole. The price of etofenprox had little impact on the overall unit costs (Fig. 2). Estimates of the cost of distribution of VSPR alone were 16.45 USD per pack (a nearly 60% reduction compared to distribution of the full forest pack). Distribution of topical repellents alone were estimated to cost 28.45 USD (a nearly 30% reduction). Spraying of clothing alone was estimated to cost 6.46 USD (85% reduction) per recipient per round. Operation of the three distribution systems independently might then have cost approximately 51.39 USD in total (per pack) to deliver the same quantities of the three interventions. This indicates that the combination of the three interventions into a single forest pack, led to potential cost-savings of 11 USD or approximately 21% compared to operating all three distribution systems independently.

While not examined in this study, delivering a full malaria season worth of products during one distribution round might realize savings, but such savings would also be small considering that the product constitutes nearly 90% of total cost. A shift to a low cost and long duration VPSR as the sole product in the pack could reduce costs substantially (nearly 90%) largely due to the removal of expensive topical repellents from the pack but also due to the need to deliver packs only once per season (Long Duration VPSR Only, Fig. 2). The sensitivity analysis also examined the impact of the provision of clothing with the etofenprox treatment (Price Clothing, Fig. 2) and if there was higher uptake or more effective delivery of the etofenprox treatment (Uptake Pyrethroid Treatment, Fig. 2).

### Budget impact

The budget impact of scaling the intervention to two operational districts was estimated using the cost per eligible person and multiplying by estimates of the eligible population in two operational districts [22] The budget was nearly an order of magnitude different between the base-case scenario and the lowest-cost alternative scenario for the reasons described above (Table 5). The overall budget impact is also highly sensitive to the determination of the eligible population since the forest-exposed population can vary highly by district.

**Table 5:** Estimated budget requirement to cover entire at-risk population for each operational district (USD)

Province, District	Total Population	Estimated at-risk population in district	Total Cost—Base Case Scenario	Total Cost—Low Cost Long Duration VPSR Only	Cost per Capita (Whole District)
Kampong Speu, Phnom Srouch	9562	1945	269,013	27,386	28.13
Mondulkiri, Sen Monorom	26,026	17,315	2,394,838	243,795	92.02

## Discussion

This study assessed the costs of delivering bite prevention products in a forest pack to forest exposed populations in Cambodia, estimating a total cost per eligible person of approximately 138 USD [43 USD per person for the entire population of targeted areas (where only the high-risk sub-population receives the intervention)]. This estimate is highly sensitive to the costs of the individual products and reflects product costs at current pricing, which is expected to decrease when scaled for the public health market, potentially with market shaping efforts.

In the context of Project BITE, the topical repellents and etofenprox solution were donated, but, at reasonable market prices used in this costing study, all three products would contribute nearly 90% of the total cost of the intervention. The cost of *delivery* of these products contributes very little to the overall cost of the intervention with the total and unit costs being driven largely by the prices of topical and spatial repellent products. While this analysis followed a provider perspective and thus excluded the value of clothing brought for treatment by eligible participants, inclusion of reasonable costs for this clothing did not add significantly to the overall cost of the intervention. This was due largely to the low price and small quantities of etofenprox required for the treatment and the relatively low uptake of this component of the intervention – 50% of the intended treatments were not conducted due in large part to logistical challenges, including bulky packaging and challenges with getting clothing provided by participants as well as perceived risks. User experience with the products was evaluated in depth and is described in a separate manuscript in preparation. Even with higher uptake examined under sensitivity analysis, the costs were expected to remain relatively low for etofenprox treatment.

While the cost of USD 138 per eligible person for a four-month season is high compared to the cost of distribution of household-focused vector control tools (*i.e.*, indoor residual spraying and insecticide-treated bed nets), few bite prevention products are available to provide protection from mosquito bites for this population outdoors. To our knowledge no studies of the cost of delivering supplementary malaria prevention products to this population have been conducted [23, 24]. The VPSR used in this study, BiteBarrier, was a prototype design and was costed based on anticipated consumer market price. Work is ongoing with multiple manufacturers to create pipelines for VPSRs priced for public health and humanitarian scenarios, with anticipated prices much lower than the costs included in the present study. The price of high-quality topical repellents must also decrease to enable greater access. “Last mile” interventions as countries near elimination may carry higher costs per person at risk as the denominator shrinks and more complex combinations of interventions are needed to eliminate remaining foci of transmission.

Because of the high costs of products relative to their distribution, limited cost savings is made by integrating all three products into a forest pack. Integration, however, may have delivered cost savings as large as 10–20% at these product prices, and perhaps larger relative cost savings would be possible at lower product prices. It is also possible that the VPSR or topical repellent might act as complements to the other products. Being a complementary product means that the use or uptake of these products might be positively influenced by the inclusion of the other product. Pyrethroid treatment of clothing did not appear to be a complementary product in this context since its uptake was generally poor. That being said, it is also possible that the uptake of pyrethroid clothing treatment might have been even lower had VPSR or topical repellents not been included. Uptake could have been low due to factors unrelated to the attractiveness of the etofenprox treatment, but rather to distribution issues which, if addressed, could improve the cost profile of the forest pack alongside uptake of etofenprox treatment. Studies on user experience revealed relatively high acceptability of the clothing treatment if treatment was applied, suggesting low reach was more of a delivery challenge. A complementary discrete choice experiment [20] suggests that VPSR was the most favoured of the three products of the forest pack and showed the highest willingness to pay on the part of study participants.

Budget impact analysis showed that the entire forest pack delivered in this setting would likely be unaffordable at current composition and prices but that foreseeable changes to the pack composition and product prices could bring the overall budget impact to much more reasonable levels. The costs of products are expected to decrease over time, but proactive efforts should be made to rapidly develop public health and humanitarian pricing to meet growing demand, which would dramatically reduce the cost of the forest pack. Improved targeting to the highest risk populations might also improve value for money from these interventions and affordability if such targeting could be done at relatively low cost. A modelling study conducted as part of Project BITE reveals that impact is greater with effective targeting of the products to those at highest risk of mosquito bites and malaria exposure [25].

This analysis did not consider malaria or other health outcome data and thus the cost-effectiveness of the products cannot be calculated. This also prevents the examination of possible synergistic or antagonistic effects of the three products here in terms of bite prevention or health outcomes.

## Conclusions

Bite prevention products can be delivered in a forest pack to forest-exposed, malaria high-risk populations in Cambodia. At current product prices, it would cost approximately 138 USD per eligible/targeted person to distribute four months’ worth of forest packs (one malaria season’s worth in this context). This cost is almost entirely driven by product prices, and further reductions in the prices of VPSRs and topical repellents may be necessary to make distribution of these products a financially viable strategy at scale for these populations. The cost of pyrethroid treatment of clothing was relatively low, but the uptake of this intervention was also low. Because of the high costs of products relative to their distribution, little relative cost savings are made by integrating all three products, though total costs of distribution would be higher if each product were distributed independently. This costing study may help the Cambodia national malaria programme and its partners plan for further deployment of these bite prevention tools and guide efforts in other elimination settings in the GMS and beyond.

## Data Availability

All research data can be made available upon request.

## Abbreviations

ETO: Etofenprox
GDP: Gross domestic product
GMS: Greater Mekong subregion
IRS: Indoor residual spraying
ITN: Insecticide-treated bed net
MMW: Mobile malaria worker
SBCC: Social and behaviour change communication
SR: Spatial repellent
TR: Topical repellent
USD: United States Dollar
VMW: Village malaria worker
VPSR: Volatile pyrethroid spatial repellent
